# Fabrication and assessment of ethosomes for effective transdermal delivery of loxoprofen

**DOI:** 10.22038/ijbms.2025.84183.18206

**Published:** 2025

**Authors:** Sarah Jabbar Abd Alhur, Hasanain Shakir Mahmood

**Affiliations:** 1 Department of Pharmaceutics, College of Pharmacy, Mustansiriyah University, Baghdad, Iraq; 2 Department of Pharmaceutics, College of Pharmacy, University of Kerbala, Kerbala, Iraq; 3 Department of Pharmaceutics, College of Pharmacy, University of Alkafeel, Najaf, Iraq; 4 ARCPMS, University of Alkafeel, Najaf, Iraq

**Keywords:** Ethosomes, Loxoprofen, Nanocarriers, NSAID, TDDS

## Abstract

**Objective(s):**

To formulate and evaluate ethosomes for the transdermal delivery of loxoprofen, a potent non-steroidal anti-inflammatory drug (NSAID).

**Materials and Methods:**

Fifteen ethosomal formulations were created via thin-film hydration and probe sonication techniques, with variations in the amounts of egg yolk lecithin, ethanol, cholesterol (CHOL), Tween 80 (TW80), and propylene glycol (PG). The formulations were assessed for their particle size (PS), zeta potential (ZP), polydispersity index (PDI), pH, and entrapment efficiency (EE). Field scanning electron microscopy (FSEM) was utilized to evaluate their morphology. The *in vitro* drug release and *ex vivo* permeability of the ethosomal formulations were evaluated against those in a hydroethanolic drug solution.

**Results:**

The formulation labeled F14, comprising 1% loxoprofen, 1% egg yolk lecithin, 30% ethanol, 5% propylene glycol, and phosphate-buffered saline (PBS) up to 25 ml, was recognized as an optimized ethosomal formulation. These ethosomes demonstrated an average size of 164.2±19 nm, a PDI of 0.280±0.028, a ZP of +45.1±4.5 mV, and an EE of 96.8±0.43%. *In vitro* and *ex vivo* tests demonstrated that the ethosomal formulation (F14) showed superior drug release and penetration rates compared to a conventional hydroalcoholic solution. The differential scanning calorimetry (DSC) study showed that loxoprofen was completely trapped within ethosomes. On the other hand, the Fourier transform infrared (FTIR) study confirmed that the drug and the additives did not interact.

**Conclusion:**

The current study revealed that loxoprofen can be effectively delivered transdermally via the ethosomal system.

## Introduction

**Figure 1:**
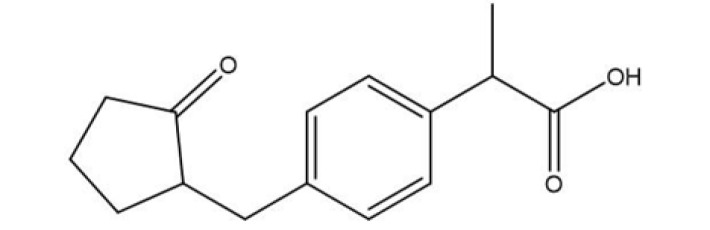
Chemical structure of loxoprofen

Loxoprofen, a prodrug categorized as a short-acting non-steroidal anti-inflammatory drug (Figure 1), requires metabolic conversion to its active form predominantly in the liver, with partial conversion occurring in the bloodstream and the skin’s dermal layer (1). Despite its therapeutic potential, repeated oral administration of loxoprofen has been associated with significant gastroduodenal mucosal damage and upper gastrointestinal bleeding. However, unlike many other NSAIDs, loxoprofen demonstrates a favorable cardiovascular safety profile, suggesting its suitability for transdermal delivery as a promising alternative (2). The Transdermal drug delivery system (TDDS) represents a practical method wherein the drug permeates the systemic circulation through the skin, which serves as the primary target for topical and transdermal formulations. This method reduces gastrointestinal adverse effects and improves patient adherence. Nevertheless, the inherent barrier function of the stratum corneum (SC) complicates the attainment of significant skin penetration with conventional transdermal formulations, thereby restricting the drug’s effectiveness. Numerous approaches have been investigated to improve drug penetration rates, with the vesicular system being especially significant. Vesicular lipid systems, such as ethosomes, represent one of the most promising approaches in transdermal drug administration (3). In 1996, Touitou *et al*. first conceived and constructed ethosomes. Ethosomes are systems made up of flexible vesicles primarily composed of phospholipid, ethanol at a relatively high concentration (20-50%), and water (4). Among all phospholipids, lecithin is the preferred phospholipid to formulate ethosomes (5). Ethanol serves as an efficient skin permeation enhancer by disrupting lipid organization in the stratum corneum and extracting its lipid layers, resulting in decreased lipid density and reduced skin impermeability to permeants. Also, Ethanol-induced fluidization of lipid bilayers in the stratum corneum enhances the penetration of small, flexible ethosomes into deeper skin layers, where they amalgamate with skin lipids and release their contents (6). Moreover, ethanol enhances the flexibility, deformability, and elasticity of ethosomes compared to traditional liposomes, hence augmenting their capacity to permeate various skin layers. These effects render ethosomes an innovative drug delivery system for transdermal administration (7). This research intends to formulate and characterize ethosomal preparations for the transdermal administration of loxoprofen. Specifically, it investigates the effects of ethanol, lecithin, and additives on manufactured ethosomes.

## Materials and Methods

### Materials

Loxoprofen, egg yolk lecithin, and cholesterol were purchased from Meryer, China; propylene glycol was sourced from Jin Ming Biotechnology Co., Ltd., China; phosphate buffer saline (pH 7.4) and Tween 80 were procured from HiMedia Laboratories Pvt. Ltd., India; and ethanol was obtained from Sigma-Aldrich, USA. All chemicals and materials utilized for the physical creation of ethosomes were of analytical grade. Distilled water was utilized consistently during the experiment.

### Animal experiment 

The male albino rats, weighing 200 to 250 gr and aged 8 to 10 weeks, were sourced from the animal house of College of Pharmacy at the University of Kerbala, Iraq. They were utilized for a skin irritation investigation and an *ex vivo* permeation study of the ethosomal formulation. The study protocol received approval from the ethical committee at the College of Pharmacy, Al-Mustansiriya University.

### Methods

#### Preparation and optimization of loxoprofen containing ethosomes

Ethosomes were formulated employing the thin film hydration method, in which a lipid mixture of egg yolk lecithin, cholesterol, and loxoprofen powder was dissolved in 10 ml of ethanol within a 500 ml round-bottom flask and subsequently evaporated using a rotary evaporator (Heidolph, Germany) at 45 ^°^C for one hour, yielding a thin, dry lipid film on the flask wall. The film was subsequently rehydrated by including a mixture of ethanol and phosphate-buffered saline (pH 7.4) in different ratios with continuous agitation, followed by homogenization using sonication (Hielscher, Germany) at 200W for two cycles of 5 min each (8). The formulations were prepared as detailed in Table 1; the values and concentrations in this table were derived from a thorough review of published studies on similar formulations (9-12). Various independent variables were evaluated to enhance the ethosomal formulations of loxoprofen concerning PS, ZP, PDI, and EE, whereby five factors were ultimately chosen for a total of 15 formulations: ethanol concentration, egg yolk lecithin concentration, CHOL concentration, TW80 concentration, and PG concentration (13). 

### Evaluation of ethosomal formulations

Evaluation of vesicle size, size distribution, and zeta Potential 

Dynamic light scattering (DLS) was used as a computer-assisted analysis method to evaluate the PS, ZP, and PDI of the ethosomal solution (14). Before measurements, 1 ml of the ethosomal suspension was diluted to 10 ml with a hydroethanolic mixture using a Zetasizer apparatus (Microtrac, USA). These measurements were performed under controlled conditions at 25 ^°^C (15).

### Determination of entrapment efficiency

The estimation of loxoprofen entrapped within the ethosomes involved the removal of the unentrapped drug. The dispersion was centrifuged in a cooling centrifuge (Hitachi, Germany) at 12,000 rpm and 4 ^°^C for one hour, separating ethosomes pellets and a supernatant containing the free active ingredient. Following suitable dilution with phosphate buffer solution, the supernatant was analyzed for drug concentration by measuring absorbance at 223 nm using a UV-VIS spectrophotometer (Shimadzu, Japan). The encapsulation efficiency percentage of all ethosomal formulations was computed using the equation:

$\mathrm{EE\%}=\frac{\mathrm{A1}-\mathrm{A2}}{\mathrm{A2}}\times 100$

A2 represents the amount of loxoprofen initially added and the amount determined in the filtrate by spectrophotometry, respectively, and A1-A2 represents the amount of loxoprofen included in the formulation (16).

### pH measurement 

The pH of each ethosomal formulation was measured using a pH meter (HQ11D, HACH, USA) to ensure compatibility with the skin’s pH (17). 

### In vitro drug release study

For the *in vitro* drug release study, a dialysis bag with a molecular weight cutoff of 8,000 to 14,000 Daltons was used to stop the drug from moving between the donor and receptor compartments. Ethosomal formulations (60 mg) encased in dialysis bags were immersed in distinct vials containing 100 ml of PBS (pH 7.4) supplemented with 1% ethanol, maintained at 37 ^°^C with horizontal agitation at 100 rpm. Samples were collected at 15, 30, 60, 120, 180, 240, and 300 min, with fresh medium added at each interval. The collected samples were subsequently tested for active component content using the UV-visible technique. The drug release properties of loxoprofen-ethosomes were assessed during three repetitions of the experiment and compared to the hydroethanolic solution of loxoprofen (18). The results of *in vitro* drug release experiments were analyzed to understand the kinetics of drug release from the ethosomal formulation using several mathematical kinetic models, such as zero-order kinetics, first-order kinetics, the Korsmeyer-Peppas model, and the Higuchi model. The D.D. solver program integrated into Microsoft Excel 2016 was employed to examine the release kinetic models. The optimal model was selected based on the highest R-squared values among the four models (19). 

### Selection of the optimum ethosomal formula

The ethosomal formulation to be identified as optimal should comply with the requirements of particle size under 200 nm, narrow size distribution, zeta potential exceeding +30 mV, entrapment efficiency surpassing 90%, and rapid *in vitro* drug release percentage (10). Rapid drug release indicates the carrier system’s ability to efficiently release the drug into the skin layers, a critical step in ensuring therapeutic efficacy.

### Characterization of optimum ethosomal formula 

Vesicular morphology and surface topology 

*The morphology, size, and size distribution of the lyophilized ethosomal formulation were examined using FSEM (FEI COMPANY, USA). Before examination, the material was affixed to copper stubs using double-sided tape, subsequently coated with gold, and then examined at various magnifications (* 20 *).*

### Skin irritation study

The study involved three groups of male albino rats (n=3 per group): (1) untreated control, (2) treated with the drug solution, and (3) treated with the optimized ethosomal formulation. Prior to application, the rats’ skin was depilated 24 hr in advance. Optimized ethosomal suspension containing 60 mg of the medication was administered to the depilated skin of the rats. Thereafter, the skin surface was evaluated at 1, 24, 48, and 72 hr post-application for observable alterations, particularly erythema. Erythema scores were allocated according to the observed severity: no erythema (0), mild erythema (1), moderate erythema (2), moderate to severe erythema (3), and severe erythema (4)(21). 

### Ex vivo permeation assessment

*Ex vivo* investigations were conducted utilizing the skin of albino rats, employing Franz diffusion cells with an absorption area of 1.77 cm² and a receptor cell volume of 50 ml. The receptor compartment medium consisted of phosphate buffer at pH 7.4 combined with 1% ethanol, kept at skin temperature, and continuously agitated by a magnetic stirrer at 100 rpm during the experiment. In this study, the rat skin was positioned in a Franz diffusion cell, with the stratum corneum facing upwards towards the donor compartment and the dermis facing downwards against the receptor compartment. A sample of optimized ethosomal formulations containing 60 mg of loxoprofen was administered to the skin surface. Samples were collected at designated intervals over eight hours and replaced with fresh buffer solution to maintain sink conditions. Each trial was executed thrice. The cumulative quantity of loxoprofen that traversed during each time interval, the steady-state flow rate (J), the lag time, the apparent permeation coefficient (Papp), and the enhancement ratio (ERp) were all calculated. The enhancement ratio was computed utilizing the provided equation:

${\mathrm{ER}}_{p}=\frac{K_{\mathrm{p1}}}{K_{\mathrm{p2}}}$

Kpl denotes the observed permeability coefficients of the selected formula, whereas Kpo signifies the observed permeability coefficients of the control (22). 

### Thermal examination of optimal loxoprofen-loaded ethosomes Formula

DSC (Shimadzu, Japan) was used to conduct thermal analysis on various samples, including loxoprofen, egg yolk lecithin, polymer, a physical mixture of loxoprofen, and egg yolk lecithin and optimized lyophilized ethosomal formulation. The samples were positioned on an aluminum pan, sealed, and perforated with pinholes. The samples were subsequently heated from 25 to 200 ^°^C under nitrogen at 10 °C/min, and the heat flow was documented. The data was plotted on a graph with temperature on the X-axis and heat flow (w/min) on the Y-axis (23). 

### Drug-excipient compatibility study

Drug-excipient compatibility is examined via Fourier transform infrared spectroscopy (BRUKER, Germany) with a detector cell that scans between 4000 and 400 cm⁻¹. The optimized lyophilized ethosomal formulation and its physical mixture were evaluated and compared with the pure drug using FTIR (24). 

### Assessment of stability

The stability of the optimized formulation was evaluated by storing samples in tightly sealed vials at 4 ^°^C for three months. After three months, the ethosomal carriers were visually examined for any physical alterations. Subsequently, the vesicles’ size, ZP, pH, and EE were analyzed (25).

### Statistical analyses

Statistical analyses were performed utilizing Prism Version 9 (GraphPad Software, San Diego, CA, USA). All data are expressed as means±standard deviation. The statistical analyses comprised t-tests, one-way ANOVA, and two-way ANOVA, contingent upon the quantity of independent variables. Tukey’s test was employed for *post hoc* analysis to ascertain significant group differences. A *P*-value below 0.05 was deemed indicative of statistical significance.

## Results

### Preparation and optimization of loxoprofen containing ethosomes

Fifteen ethosomal formulations were created via thin-film hydration and probe sonication techniques, with variations in the amounts of egg yolk lecithin, ethanol, CHOL, TW80, and PG. All developed formulas were analyzed and assessed for optimization.

### Evaluation of ethosomal formulation

The study evaluated the characteristics of ethosomal formulations, encompassing PS, PDI, ZP, pH, and EE, as reported in Table 2. The vesicle sizes in the ethosomal formulations (F1-F15) ranged from 164.2±19 nm to 336.2±154.6 nm. ZP ranged from 30.9±3.8 mV to 70.8±4.2 mV, indicating their physical stability under the specified temperature conditions. A PDI falling within the range of 0.1 to 0.3 indicates a uniform distribution and narrow size range, while values exceeding 0.3 signify a heterogeneous distribution. According to the results, all formulations’ pH values closely matched normal skin’s pH range (4.5-6.5). Also, the entrapment efficiency of ethosomes was assessed across all formulations, revealing a minimum of 72.35±2.88% for formulation F7 and a maximum of 96.98±0.43% for formulation F14. 

### In vitro drug release studies

**Figure 2:**
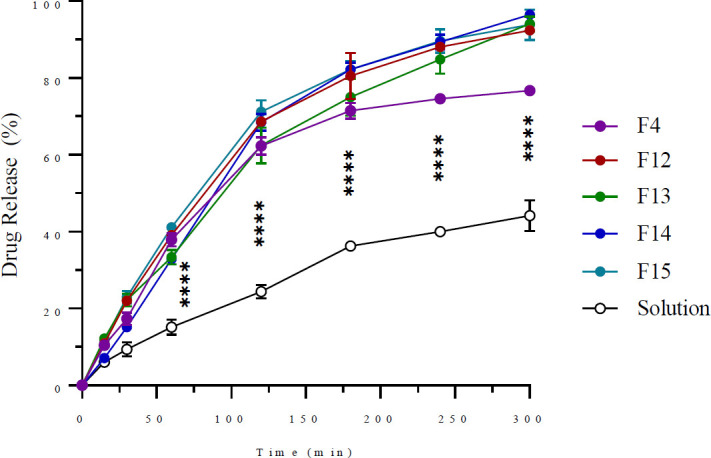
*In vitro* release from various ethosomal formulations

Formulas (F4, F12, F13, F14, and F15) were selected for the release test based on particle size and entrapment efficiency, prioritizing formulations with smaller particle sizes and higher EE values. All ethosomal suspensions exhibited comparable release profiles, characterized by an early rapid release of chemicals from the ethosomes followed by a constant release. In addition, the ethosomal formulation releases more medication than hydroethanolic solutions, as depicted in Figure 2. According to Table 3, the release kinetics study shows that the release of ethosomal formulations follows a first-order kinetic model, and the drug solution follows a Korsmeyer-Peppas model.

### Selection of the optimum ethosomal formula

The study results found that the F12, F14, and F15 formulations matched the criteria for selecting the optimum ethosomal formula. However, F14 has a smaller particle size than F12 and F15 formulations. The effective entrapment of drugs and the *in vitro* drug release are similar. Therefore, among the fifteen formulas (F1-F15), F14 can be considered the optimum formula. This formulation gave ethosomes with 164.2±19 nm vesicular size (PDI=0.28±0.028), ZP of 45.1±4.5mV, EE of 96.98±0.43% and drug release of 96.47±0.65%. Accordingly, it was selected for further studies.

### Characterization of optimum ethosomal formula 

*Vesicular shape and surface morphology*

**Figure 3:**
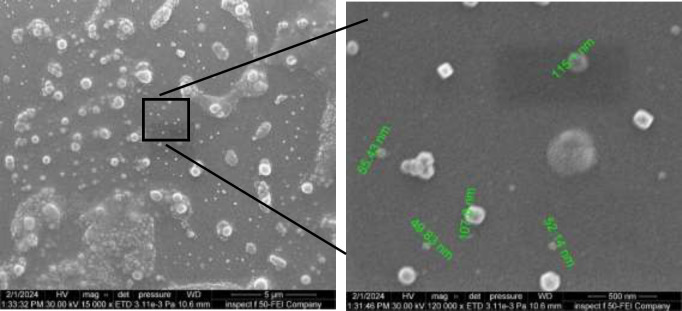
Field emission scanning electron microscopy (FESEM) images of the optimized ethosomes formulation (F14)

**Figure 4:**
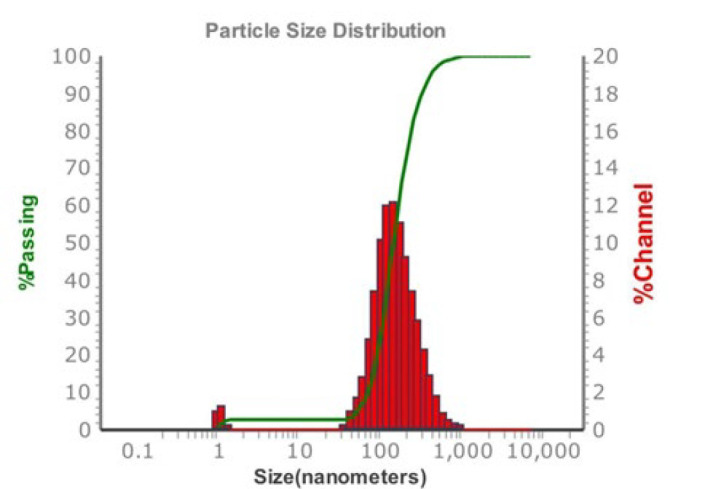
Particle size distribution of optimum ethosomal formula (F14)

 FESEM images of the optimized ethosomes formulation (F14), as depicted in Figure 3, revealed the existence of smooth, diminutive, spherical vesicles exhibiting a homogeneous distribution. The visuals correspond well with the particle size values acquired from the analyzer, as depicted in Figure 4.

### Skin irritation study

**Figure 5:**
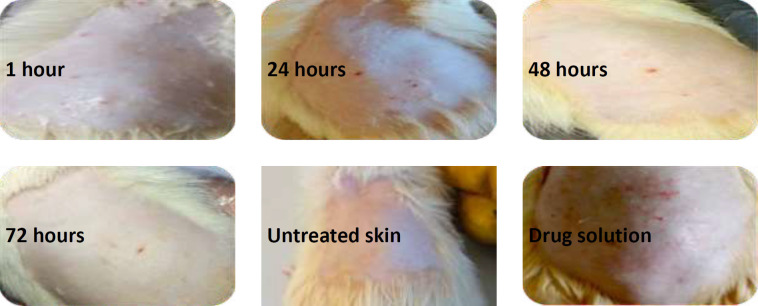
Images of the skin irritation test performed by applying the optimized ethosomal formulation (F14) on the rat’s skin at 1, 24, 48, and 72 hr, along with images of the untreated area and the area treated with the drug solution

The skin response to the optimized loxoprofen-ethosomal formula (F14) was assessed through an acute irritation test in rats, focusing on the formation of erythema, eschar, and edema. After 72 hr, application of the loxoprofen-ethosomal formula to the test rats showed no signs of erythema, eschar, or edema (score = 0), as shown in Figure 5. 

### Ex vivo permeation assessment

**Figure 6:**
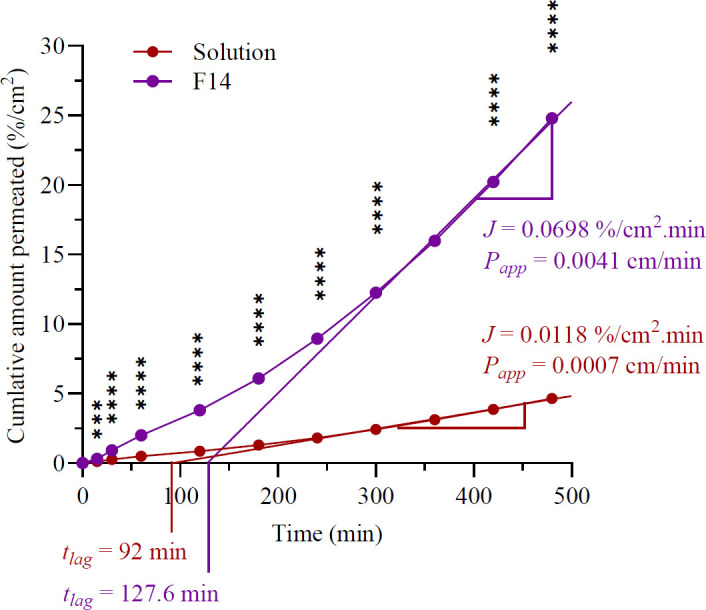
Cumulative percentage permeated per unit area over time for F14 and solution formulations

Figure 6 illustrates the outcome of the *ex vivo* permeation investigation, wherein the optimized ethosomal formulation (F14) had almost complete drug permeation with a percentage of 95.23%. In comparison, the hydroethanolic solution of the medication showed 18.9 % drug permeation after the same time. The steady-state flux (Jss) and apparent permeability coefficient (Papp) were also determined for the tested preparations. Both parameters followed this descending order: optimized ethosomes> drug solution. Also, the lag time for ethosomes is longer (127.6 min) than the hydro-alcohol solution (92 min). 

### Thermal examination of optimal loxoprofen-loaded ethosomes Formula

**Figure 7:**
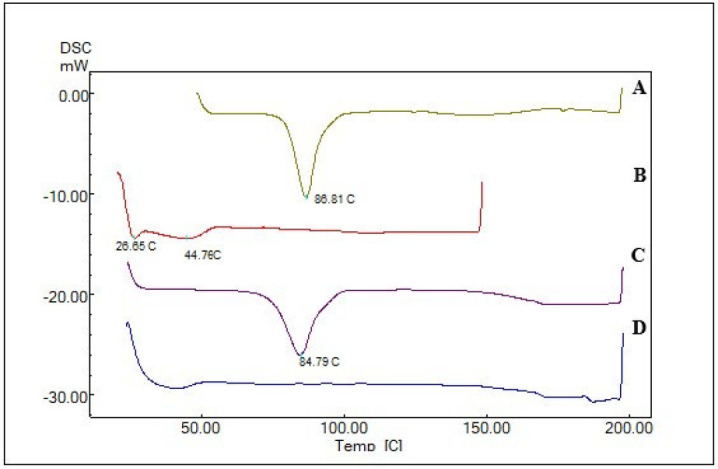
Differential scanning calorimetry (DSC) thermograms of pure loxoprofen (A), egg yolk lecithin (B), physical mixture of loxoprofen and egg yolk lecithin (C), and optimized formulation of ethosomes (D)

Figure 7 shows the DSC thermogram of pure loxoprofen, egg yolk lecithin, physical mixture, and optimized formulation of ethosomes (F14). The DSC thermogram of pure loxoprofen displayed an endothermic peak at 86.8 °C (Figure 7A), which was absent in the thermogram of the optimized ethosomal formulation (Figure 7D). 

### Drug-excipient compatibility study 

**Figure 8:**
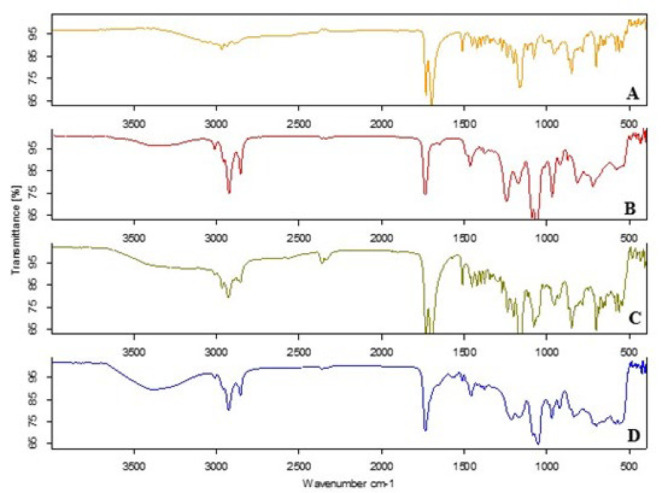
Fourier transform infrared (FTIR) spectrums of pure loxoprofen (A), egg yolk lecithin (B), physical mixture of loxoprofen and egg yolk lecithin (C), and optimized formulation of ethosomes (D)

Figure 8D illustrates the FTIR spectra of the optimized ethosomal formulation (F14), providing insights into the compatibility between the drug and excipients. The FTIR spectrum of loxoprofen (Figure 8A) exhibits several prominent peaks: a broad O-H stretching peak between 2540–3400 cm⁻¹, C=O stretching peaks at 1698.95 cm⁻¹ and 1731.38 cm⁻¹, aromatic C-H stretching peaks near 2966 cm⁻¹, aromatic C=C stretching peaks around 1465–1533.57 cm⁻¹, alkyl C-H stretching peaks approximately at 2935 cm⁻¹, and C-H bending peaks between 1350–1470 cm⁻¹. The FTIR spectrum for egg lecithin (Figure 8B) exhibits its main characteristic bands as follows: broad O-H and N-H stretching peaks around 3367 cm⁻¹, C-H stretching peaks around 3000 cm⁻¹, a sharp C=O stretching peak around 1735 cm⁻¹, a P=O stretching peak around 1243 cm⁻¹, P-O-C stretching peaks around 1089 cm⁻¹, C-H bending peaks around 1465 cm⁻¹ and 1376 cm⁻¹, and N+(CH3)3 stretching peaks around 3009.93 cm⁻¹**. **

### Assessment of stability

**Figure 9:**
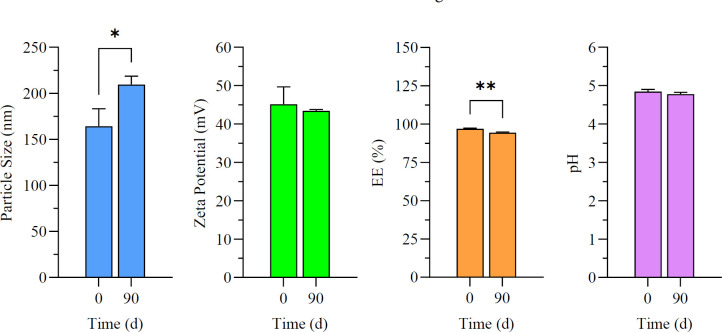
Impact of long-term storage (90 days) on the particle size, zeta potential, entrapment efficiency, and pH of the ethosomal formulation (F14) at 4 ^°^C

The physical stability of the ethosomal solution (F14) was assessed over 90 days at 4 °C by measuring parameters including size, pH, entrapment efficiency (EE), and zeta potential. On day 90, there were significant changes (*P*<0.005) in the particle size and ethosomes’ ability to trap particles (Figure 9). 

## Discussion

This study focused on formulating and assessing an ethosomal preparation for effective transdermal delivery of loxoprofen. The formulations displayed white colloidal suspensions without any precipitation of free medication, indicating that the incorporation of ethanol as a co-solvent, together with micelle solubilization of phospholipids, may produce a synergistic effect. This action can improve the solubility of loxoprofen in the formulation. Formulation (F4), including 1% w/v loxoprofen, 1% w/v egg yolk lecithin, and 30% v/v ethanol, was selected as the primary component for future development owing to its smaller particle size. Supplementary components, including CHOL, TW80, and PG, were added to improve the entrapment efficiency and stability of the loxoprofen-ethosomal formulation. Cholesterol (F10-11) serves as a stabilizing factor by inhibiting leakage and diminishing the membrane permeability of vesicles. This leads to an augmentation in both the dimensions of the vesicles and their capacity to encapsulate drugs effectively. In addition, Tween80 (F12-13) can improve both the trapping effectiveness and the particles’ size due to its solubilizing properties (10). While the inclusion of propylene glycol (F14-15) in ethosomal formulations significantly enhances their stability by preventing aggregation and maintaining smaller, more uniform vesicle sizes. This effect helps maintain the structural integrity of the ethosomes over time (11). 

The size of vesicles significantly influences the delivery of pharmaceuticals. Vesicles smaller than 300 nm are considered adequate for the targeted delivery of medication to deeper skin layers, to some extent. Optimal transdermal drug delivery is attained when ethosomes dimensions are below 200 nm (26). The size of ethosomes containing 20% ethanol (F1) is 255.9±34 nm, which decreases to 195.4±6.5 nm upon increasing the ethanol concentration to 30% (F4) while maintaining a constant lecithin quantity. Ethanol fills the spaces between the hydrocarbon chains, resulting in a thinner vesicular membrane and smaller vesicles. Abdulbaqi *et al*. also observed that increased ethanol concentration correlates with reduced vesicle size (9). At an ethanol concentration of 40% (F7), the size of the ethosomes increases to 201.6±33.7 nm. This is attributable to the elevated concentration of ethanol, which leads to an unstable membrane in the ethosome structure and the dissolution of phospholipids in ethanol, which causes the vesicles to enlarge dramatically. Furthermore, elevating the lecithin concentration from 1% to 2% led to a moderate enlargement of the vesicles while maintaining a constant ethanol concentration (from 195.4±6.5 nm of F4 to 317.9±44.1 nm of F6) as lecithin molecules tend to clump together and create aggregates. These results are in agreement with previous publications (27, 28). 

Zeta potential serves as an indicator of the electrostatic stability within colloidal dispersions. It reflects the potential disparity between electronically charged ions and particles, a crucial factor in preventing aggregation. Generally, ethosomal vesicles exhibit a negative charge. However, in the present study, the ethosomes displayed a positive charge, contrasting with findings reported in most previous studies (19, 29)**. **Researchers have observed that an ethosomal system’s encapsulated medicine can change the vesicular charge from negative to positive. The phenomenon was ascribed to the incorporation of a carbonyl group. The carbonyl group functioned as an electron-withdrawing moiety, rendering loxoprofen-ethosomes positively charged (30).

The polydispersity index serves as a metric for the dispersion of vesicles, reflecting their homogeneity. The observed homogeneous nature of ethosomal formulations is likely attributed to the predominant surfactant activity of lecithin (31). 

The optimized formulation (F14)’s pH was 4.84±0, closely aligning with the natural skin pH range of 4.5-6.5, as observed in all other formulations. This is important for minimizing irritation, improving user comfort, and guaranteeing efficient drug administration (32). 

Entrapment efficiency is a metric associated with the delivery capability of vesicular systems. Among all vesicular systems, ethosomes are distinguished by their elevated value. The effect of ethanol concentration on the percentage of drug entrapment in ethosomes was observed, indicating an enhancement in the aqueous solubility of loxoprofen with increased ethanol concentrations, likely attributed to its co-solvent effect. Higher ethanol concentrations facilitated greater drug accommodation within the aqueous core of the vesicles, as observed in ethosomal formulation (F1) relative to other ethosomal formulations (F4). However, concentrations exceeding 30% resulted in drug leakage from the fluidized bilayer of vesicles, reducing the percent of drug entrapment of ethosomes as seen in formulation (F6) compared to formulation (F9). A similar trend was previously reported in other studies (33, 34). Moreover, entrapment efficiency increased with higher concentrations of lecithin, with formulation (F4) exhibiting 80.43±0.92% and formulation (F6) showing 95.13±0.52%. This phenomenon may be attributed to the drug’s lipophilic nature, which enables enhanced encapsulation within the lipid bilayer of the formulation. Faisal *et al*. discovered the same findings earlier, noting that the EE% of voriconazole augmented with a rise in phospholipid content (35).

*In vitro* release rates are critical indicators of the quality of transdermal drug delivery systems. The *in vitro* release of loxoprofen from different ethosomal formulations was evaluated using the dialysis membrane technique, resulting in a graph depicting the drug release percentage with time, with maximum drug release occurring after five hours. The rapid release of loxoprofen transpires via the diffusion of the drug from vesicles into the surrounding liquid across the cellophane membrane. Elevated ethanol concentrations enhance vehicular mobility and the fluidity of ethosomal vesicle bilayers. Consequently, all ethosomal suspensions exhibited comparable release profiles, characterized by an early rapid release of chemicals from the ethosomes (0-60 min), followed by a constant release over hours (120-300 min). This indicates that ethosomes can provide controlled drug delivery (36). Additionally, it was shown that the elevated release rate (*P*<0.0001) from the optimized ethosomal formulation (F14)(96.47±0.65%) compared to the ethosomal formulation (F4) (76.71±0.18%) might be attributed to the inclusion of an alcohol combination in the former, as opposed to ethanol alone in the latter, consistent with other research (37). This may enhance drug solubilization in the ethosomes, resulting in expedited drug release. The enhanced deformability of the vesicle bilayer resulting from the presence of PG may have also facilitated a higher drug release rate. Ultimately, the *in vitro* drug release from ethosomes (76.71±0.18-96.47±0.65%) is superior to that from hydroethanolic solutions (44.13±4.06%). This contradicts earlier published reports (38, 39). The exact cause of this discrepancy remains unclear; however, it could be attributed to their unique vesicular architecture, which increases bilayer membrane fluidity due to ethanol content. This structural feature and improved solubility likely promote greater drug release. The elevated medication release rate of the nanocarriers may be beneficial for *in vivo* applications to attain expedited therapeutic effects (37, 40). 

The D.D. solver in Microsoft Excel 2016 examined the data from *in vitro* drug release using four kinetic models. The model selection was based on several criteria, including adjusted R²; the highest adjusted R² value indicates the best-fitting model. The release kinetics analysis results reveal that the release follows a first-order kinetic model with an R² value of 0.9829 for optimal ethosomal formulation (F14). Further analysis using the Korsmeyer-Peppas equation yielded a release exponent (n) of 0.661. Based on n values, systems with 0.45<n<0.89 have non-Fickian diffusion and erosion as the release mechanism (36).

After 72 hr, application of the optimized loxoprofen-ethosomal formulation to the test subjects showed no signs of erythema, eschar, or edema (score=0). Consequently, the absence of erythema after 72 hr can be ascribed to the improved encapsulation efficacy of the medication, potentially diminishing irritation. Increased entrapment efficiency in ethosomes indicates that a more significant percentage of the drug is contained inside the vesicles, resulting in fewer unentrapped drug molecules in the formulation. This is advantageous for alleviating skin irritation since free medication molecules may come into contact with and irritate the skin. In contrast, the drug solution caused moderate erythema, limiting its usability and acceptance. Thus, ethosomes containing loxoprofen could minimize irritation and enhance patient compliance (21). 

This study aimed to evaluate the quality of the formulation in facilitating drug permeation through the skin upon application, as this process directly impacts therapeutic efficacy. The assessment was conducted using excised rat skin. The outcomes of the *ex vivo* permeation test revealed that the ethosomal vesicles exhibited a significantly higher cumulative percentage of permeated and enhanced drug permeation compared to the hydroethanolic drug solution. Specifically, the steady-state flux of the ethosomal formulation (F14) and the hydroethanolic drug solution was recorded as 0. 0698%/cm².min and 0118%/cm².min, respectively. Additionally, the permeability coefficients for F14 and the drug solution were 0.0041 cm/min and 0.0007 cm/min, respectively, after eight hours. These findings suggest that the optimized ethosomal formulation demonstrated approximately 7-fold and 6-fold higher flux and permeability coefficients, respectively, when compared to the drug solution. This indicates a direct correlation between flux and the permeability coefficient. The observed enhancements in permeation may be attributed to the synergistic interaction between the lipid vesicles and the skin’s lipid matrix, resulting in significantly superior permeation (*P*<0.001 and *P*<0.0001) compared to the drug solution. These results confirm that the ethosomal system facilitates improved transdermal drug delivery, aligning with findings reported in other studies (37, 41). Interestingly, it takes longer time for the drug to start showing up in the receiving compartment after the first application with ethosomes than with the hydro-alcohol solution. These data demonstrate that ethosomal formulations enhance skin penetration and facilitate loxoprofen depot development within the skin. The phospholipid component of the ethosomes, which might prolong drug retention in skin tissues by vesicle fusion with skin lipids, acts as a depot reservoir (42). The results also show that optimized ethosomes had an enhancement ratio in loxoprofen Papp of 5.85 relative to the drug solution. Akhtar N. and colleagues developed a tocopherol acetate-loaded ethosomal gel formulated with 1% w/w carbopol 940, demonstrating a 1.17-fold enhancement in efficacy compared to pure tocopherol acetate (29). These findings collectively indicate that the ethosomal system significantly enhances drug permeation through the skin due to its advantageous physicochemical characteristics (43). 

DSC was utilized to investigate the physical condition of the medication enclosed within the ethosomal vesicles. The DSC thermogram of pure loxoprofen displayed an endothermic peak at 86.8 ^°^C, aligning with its documented melting point and affirming its crystalline nature (44). The thermogram of lecithin indicates a phase transition temperature of 44.76 ^°^C (39). A slight deviation in the peak (84.79 ^°^C) was noted in the thermogram of the physical mixture (1:1) of loxoprofen with egg yolk lecithin, indicating negligible or no interaction. Conversely, the DSC thermogram of the lyophilized ethosomal formulation (F14) exhibited a total absence of the loxoprofen endothermic peak. The lack of the peak indicated that loxoprofen had been dissolved into the lipid matrix and entrapped within the lipid nanocarriers (45). On the other hand, FTIR studies were conducted to examine the potential chemical interaction or stability of loxoprofen with the other components of the ethosomal formulation. After analyzing the FTIR spectra of loxoprofen and lyophilized ethosomal formula (F14), it was observed that the distinctive peaks of loxoprofen and lecithin mainly remained unaltered in both the mixture and ethosomes. Thus, the lack of notable alterations in the FTIR spectra indicates that loxoprofen and additives are compatible (46-48). 

On day 90 of storage at 4 ^°^C, the alterations in ethosomes may be ascribed to potential vesicle fusion, resulting in an increase in particle size and a diminished capacity for effective drug entrapment over time. Furthermore, the zeta potential and pH during the test do not exhibit substantial changes. Consequently, these alterations fall within permissible parameters for numerous formulations. It is crucial to evaluate the unique needs of the proposed application and potentially do long-term stability experiments to validate these conclusions (49). However, the freeze-drying technique, also known as lyophilization, removes the sample’s water content by sublimation and desorption in a vacuum, which is a way to alleviate this instability. The freeze-dried form may enhance stability by preventing particle aggregation and the loss of active compounds from vesicles (50).

**Table 1:** Compositions of loxoprofen ethosomal formulas

PBS up to (ml)	TW80 w/v%	PG v/v%	CHOL w/v%	Ethanol v/v%	Egg yolk lecithin w/v%	loxoprofen w/v%	Formula code
25				20	1	1	F1
25				20	1.5	1	F2
25				20	2	1	F3
25				30	1	1	F4
25				30	1.5	1	F5
25				30	2	1	F6
25				40	1	1	F7
25				40	1.5	1	F8
25				40	2	1	F9
25			0.5	30	1	1	F10
25			1	30	1	1	F11
25	0.3			30	1	1	F12
25	0.5			30	1	1	F13
25		5		30	1	1	F14
25		10		30	1	1	F15

CHOL: Cholesterol; PG: Propylene glycol; TW80: Tween 80; PBS: Phosphate-buffered saline

**Table 2:** Compositions of loxoprofen ethosomal formulas

Formula	PS (nm)	ZP (mV)	PDI	pH	EE (%)
F1	255.9 ± 34	35.5 ± 0.7	0.161 ± 0.107	4.81 ± 0.03	76.75 ± 0.9
F2	323.3 ± 51.6	32.5 ± 10.4	0.262 ± 0.173	4.57 ± 0.06	82.5 ± 0.26
F3	336 ± 154.6	32.5 ± 0.5	0.125 ± 0.103	4.71 ± 0.08	89.62 ± 0.38
F4	195.4 ± 6.5	43.5 ± 1	0.342 ± 0.042	4.9 ± 0.11	80.43 ± 0.92
F5	221.1 ± 51	47.2 ± 3.5	0.355 ± 0.049	4.85 ± 0.07	85.8 ± 1.87
F6	317.9 ± 44.3	45.7 ± 1.9	0.343 ± 0.18	5.09 ± 0.09	95.13 ± 0.52
F7	201.6 ± 33.7	40.4 ± 0.9	0.244 ± 0.078	5.05 ± 0.06	72.35 ± 2.88
F8	244.1 ± 44.1	44.4 ± 2.6	0.176 ± 0.081	4.8 ± 0.16	84.15 ± 1.36
F9	285.7 ± 79.5	30.9 ± 3.8	0.199 ± 0.098	4.67 ± 0.09	89.4 ± 0.71
F10	217.2 ± 13	55.3 ± 1.4	0.289 ± 0.045	4.67 ± 0.18	93.44 ± 0.79
F11	228.1 ± 10.8	49.9 ± 4.1	0.172 ± 0.026	4.85 ± 0.05	96.22 ± 0.12
F12	182.3 ± 13.3	40.6 ± 0.8	0.231 ± 0.064	4.91 ± 0.02	95.97 ± 0.41
F13	205 ± 38.5	41.9 ± 2.9	0.233 ± 0.113	4.91 ± 0.05	96.15 ± 0.61
F14	164.2 ± 19	45.1 ± 4.5	0.28 ± 0.028	4.84 ± 0.06	96.98 ± 0.43
F15	177.3 ± 9.5	70.8 ± 4.2	0.246 ± 0.049	4.56 ± 0.03	96.84 ± 0.71

PS: Particle size; ZP: Zeta potential; PDI: Polydispersity index; EE: Entrapment efficiency

**Table 3:** Release kinetic models of various ethosomal formulations (values of adjusted R²)

Formula code	Zero-order kinetic	First-order kinetic	Higuchi model	Korsmeyer-Peppas
F4	0.7716	0.9707	0.9518	0.9452
F12	0.8355	0.9979	0.9667	0.9685
F13	0.8355	0.9960	0.9753	0.9876
F14	0.8958	0.9829	0.9385	0.9599
F15	0.8202	0.9977	0.9668	0.9662
Drug solution	0.9240	0.9712	0.9656	0.9899

The data reflects the mean±standard deviation (n=3). Statistical significance is denoted by (*P<*0.0001) when comparing the drug release from several ethosomal formulations (F4, F12, F13, F14, and F15) to that of the drug solution

The graph depicts both equations’ steady-state flow (J), apparent permeability coefficient (Papp), and lag times. The data indicates the mean±standard deviation (n=3). Statistical significance is denoted by (***P<0.001, ****P<0.0001) when comparing the drug permeation from F14 to that from the solution

The study was performed with a sample size of three participants. Statistical significance is denoted by *P<0.05, **P<0.005, whereas non-significant changes (*P>*0.05) are reported

## Conclusion

In summary, the research findings indicate that the ethosomal formulation developed for enhancing the transdermal permeation of loxoprofen shows promising results. Through systematic optimization of its composition, including the careful selection of phospholipids, ethanol, and additional stabilizing agents, a formulation (F14) exhibiting favorable attributes, including reduced particle size, elevated entrapment efficiency, and improved drug release, was attained. The results indicate that the formulated ethosomal system may serve as an efficient transdermal delivery mechanism for loxoprofen, offering advantages over conventional hydroalcoholic solutions. Further investigations and clinical evaluations are warranted to confirm its therapeutic efficacy and safety for practical applications. 

## Declaration

We have not used any AI tools or technologies to prepare this manuscript.

## Future Work

This study did not utilize software such as Design Expert; however, future investigations could benefit significantly from employing such tools. Design expert can optimize formulations and experimental designs, improving precision and enhancing the overall methodological rigor of the research.
